# Integrating Metabolomics Domain Knowledge with Explainable Machine Learning in Atherosclerotic Cardiovascular Disease Classification

**DOI:** 10.3390/ijms252312905

**Published:** 2024-11-30

**Authors:** Everton Santana, Eliana Ibrahimi, Evangelos Ntalianis, Nicholas Cauwenberghs, Tatiana Kuznetsova

**Affiliations:** 1 Research Unit Hypertension and Cardiovascular Epidemiology, KU Leuven Department of Cardiovascular Sciences, University of Leuven, 3000 Leuven, Belgium; everton.santana@kuleuven.be (E.S.); evangelos.ntalianis@kuleuven.be (E.N.); nicholas.cauwenberghs@kuleuven.be (N.C.); 2 Department of Biology, University of Tirana, 1001 Tirana, Albania; eliana.ibrahimi@fshn.edu.al

**Keywords:** atherosclerotic cardiovascular diseases, metabolomics, explainable machine learning, domain knowledge

## Abstract

Metabolomic data often present challenges due to high dimensionality, collinearity, and variability in metabolite concentrations. Machine learning (ML) application in metabolomic analyses is enabling the extraction of meaningful information from complex data. Bringing together domain-specific knowledge from metabolomics with explainable ML methods can refine the predictive performance and interpretability of models used in atherosclerosis research. In this work, we aimed to identify the most impactful metabolites associated with the presence of atherosclerotic cardiovascular disease (ASCVD) in cross-sectional case–control studies using explainable ML methods integrated with metabolomics domain knowledge. For this, a subset from the FLEMENGHO cohort with metabolomic data available was used as the training cohort, including 63 patients with a history of ASCVD and 52 non-smoking controls matched by age, sex, and body mass index from the same population. First, Partial Least Squares Discriminant Analysis (PLS-DA) was applied for dimensionality reduction. The selected metabolites’ correlations were analyzed by considering their chemical categorization. Then, eXtreme Gradient Boosting (XGBoost) was used to identify metabolites that characterize ASCVD. Next, the selected metabolites were evaluated in an external cohort to determine their effectiveness in distinguishing between cases and controls. A total of 56 metabolites were selected for ASCVD discrimination using PLS-DA. The primary identified metabolites’ superclasses included lipids, organic acids, and organic oxygen compounds. Upon integrating these metabolites with the XGBoost model, the classification yielded a test area under the curve (AUC) of 0.75. SHAP analyses ranked cholesterol, 3-methylhistidine, and glucuronic acid among the most impactful features and showed the diversity of metabolites considered for building the ASCVD discriminator. Also using XGBoost, the selected metabolites achieved an AUC of 0.93 in an independent external validation cohort. In conclusion, the combination of different metabolites has the potential to build classifiers for ASCVD. Integrating metabolite categorization within the SHAP analysis further enhanced the interpretability of the model, offering insights into metabolite-specific contributions to ASCVD risk.

## 1. Introduction

Atherosclerotic cardiovascular disease (ASCVD) remains a leading cause of morbidity and mortality worldwide, largely due to complications like ischemic heart disease (IHD), stroke and peripheral arterial disease. These conditions arise from a combination of genetic predisposition, lifestyle factors (such as a poor diet, lack of exercise, and smoking), and other comorbidities like obesity, diabetes, and hypertension [1,2]. Identifying biomarkers and molecular profiles that contribute to atherosclerosis can significantly enhance diagnostic and therapeutic strategies.

Metabolomics, the high-throughput quantification of circulating metabolites in biological systems, is emerging as a critical domain in this effort, as metabolites are often reflective of real-time physiological and pathological states [3,4]. In atherosclerosis, metabolites can reveal critical insights into disease pathways, inflammatory markers, lipid profiles, and oxidative stress markers, leading to a better understanding of disease mechanisms and improving the risk stratification [5].

Previous studies have shown that metabolite-based biomarkers, particularly lipid profiles, offer added prognostic value for assessing the cardiovascular (CV) risk. For instance, McGranaghan et al. [6] showed that the cardiac lipid panel was significantly associated with fatal CV outcomes in patients with symptomatic heart failure. Other studies also demonstrated that lipid metabolites, particularly sphingolipids and ceramides, have shown promise as biomarkers for the diagnosis and prognosis of CV disease [7].

Metabolome data, however, are highly complex and often present challenges due to high dimensionality, collinearity, and variability in metabolite concentrations. Because of the complexity of the data, a metabolomic analysis often requires integrating various statistical and machine learning (ML) approaches to identify meaningful biological information and potential biomarkers. ML application in metabolomic analyses is increasing rapidly, enabling the extraction of meaningful information from complex and high-dimensional data [8]. Supervised learning methods like Random Forest, Support Vector Machines, eXtreme Gradient Boosting (XGBoost), and Partial Least Squares Discriminant Analysis (PLS-DA) are commonly used for classification tasks and feature selection, helping to distinguish between health and disease states based on metabolite profiles [9,10].

However, many ML models lack transparency, and are often termed “black box” models. Explainable ML addresses this issue by providing methods to make predictions more interpretable, which is essential in clinical contexts where understanding the reasoning behind a prediction can affect treatment decisions [11]. For tabular data, the SHapley Additive exPlanations (SHAP) method has been applied to enhance model interpretability [12,13]. Although SHAP can add information about individual metabolites, up to now, metabolites’ interactions and categorizations have not been directly integrated with explainable ML analyses.

Bringing together domain-specific knowledge from metabolomics with explainable ML methods can refine the predictive performance and interpretability of models used in atherosclerosis research. Given that domain knowledge can improve explainability and scientific consistency [14], this study has two main goals: (1) to identify the most impactful metabolites associated with the presence of ASCVD in cross-sectional case–control studies, and (2) to investigate the integration of metabolite categorization on top of the process of explainable ML by SHAP analyses.

## 2. Results

### 2.1. Characteristics of the Training Cohort

Table 1 lists the important clinical characteristics of cases and controls selected from the FLEMENGHO cohort. The two groups demonstrated similar distributions in terms of sex and BMI. However, the case group was slightly older than the control group (66 vs. 62 years old) and reported a higher prevalence of anti-hypertensive drug and statin use (Table 1).

### 2.2. Metabolites Selected by PLS-DA

The distribution of missing data is presented in Supplemental Table S2. Overall, 5 metabolites were excluded in Step 1*a*, 14 in Step 1*b* and 15 in Step 1*c*, resulting in 216 metabolites to be analyzed. The metabolites excluded in each step are listed in Supplemental Table S3.

In the training dataset, PLS-DA indicated that the optimal number of latent factors was three. These three latent factors collectively explained 64.22% of the variance between groups and 33.86% of the variance within the predictors (metabolites). This model achieved a robust performance, yielding an ROC AUC of 0.90 across the entire dataset. A total of 56 metabolites were identified as the most significant contributors to the model’s predictive capability. The complete list of the most important metabolites is given in Supplemental Table S4.

**Figure 1:**
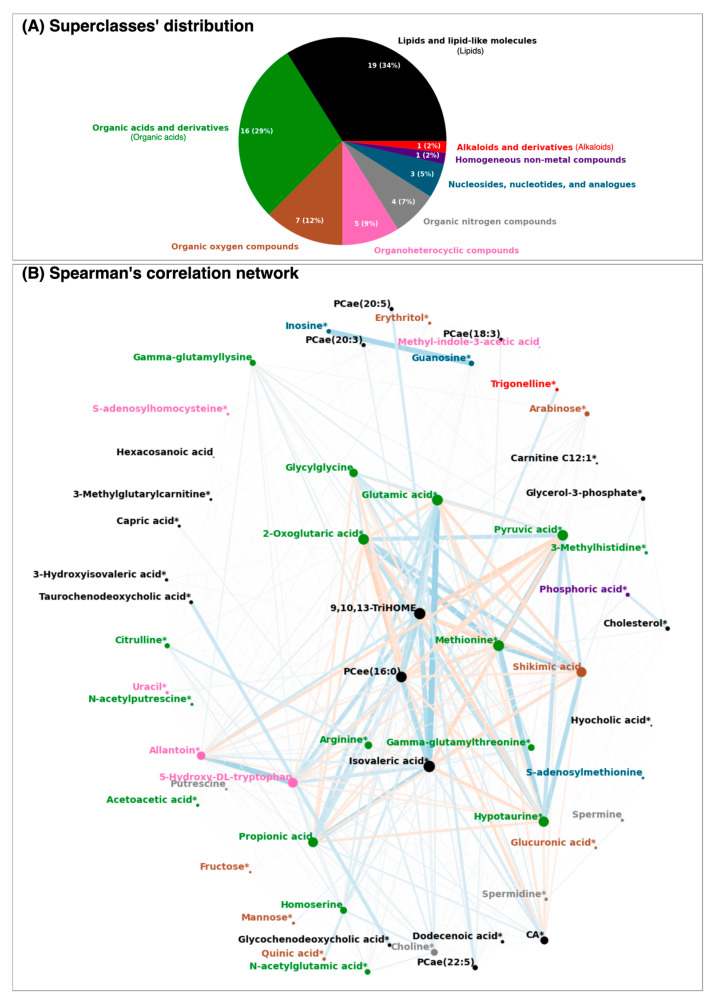
**The selected metabolites’ (A) superclass distribution and (B) Spearman’s correlation network.** In the network, the nodes correspond to the metabolites and the edges depend on the strength of their Spearman’s correlation between two nodes. Thicker and darker edges indicate a higher pairwise correlation, whereas thinner and lighter colors indicate a lower correlation. Red edges correspond to negative correlations and blue edges to positive ones. The node colors specify the metabolite superclass, and its size increases according to the absolute strength of the edges connected to it. Metabolites marked with * represent those available also in the external validation dataset. For visualization purposes, the correlations were powered to 4 but kept the original signal. In this figure, CA stands for caproic acid.

Among the 56 selected metabolites, a diverse array of metabolic pathways was represented, encompassing eight superclasses and twenty-three subclasses. As expected, the majority of these metabolites belonged to the lipids superclass, with a notable representation of fatty acids and glycerophosphocholines. Other prominent superclasses included organic acids, which were primarily composed of amino acids, peptides, and their analogues, as well as organic oxygen compounds, mainly carbohydrates, as illustrated in Figure 1A. Homogeneous non-metal compounds and alkaloids were minimally represented, with only one metabolite each, phosphoric acid and trigonelline, respectively.

The correlation network (Figure 1B) highlighted complex interconnections among metabolites, supporting the application of ML to manage these intricate interactions. Of note, the strongest correlations were not confined to metabolites within the same superclass. For example, isovaleric acid showed a strong correlation with glutamic acid, despite belonging to different superclasses. Similarly, shikimic acid displayed high correlations with diverse organic acids, such as 2-oxoglutaric acid and hypotaurine, as well as with lipids like isovaleric acid and 9,10,13-TriHOME. These cross-superclass relationships underscore the interconnected nature of the analyzed metabolic pathways.

### 2.3. XGBoost General Modeling of the Training Cohort

In the analysis of the FLEMENGHO cases and controls using the selected metabolites, the optimal hyperparameter settings for XGBoost resulted in a cross-validation ROC AUC of 0.85 ± 0.10. In the test set, the performance metrics showed an ROC AUC of 0.75 and F1-score of 0.76. The cross-validation demonstrated stability with a moderate variance. These results indicated a good performance on the test set.

**Figure 2:**
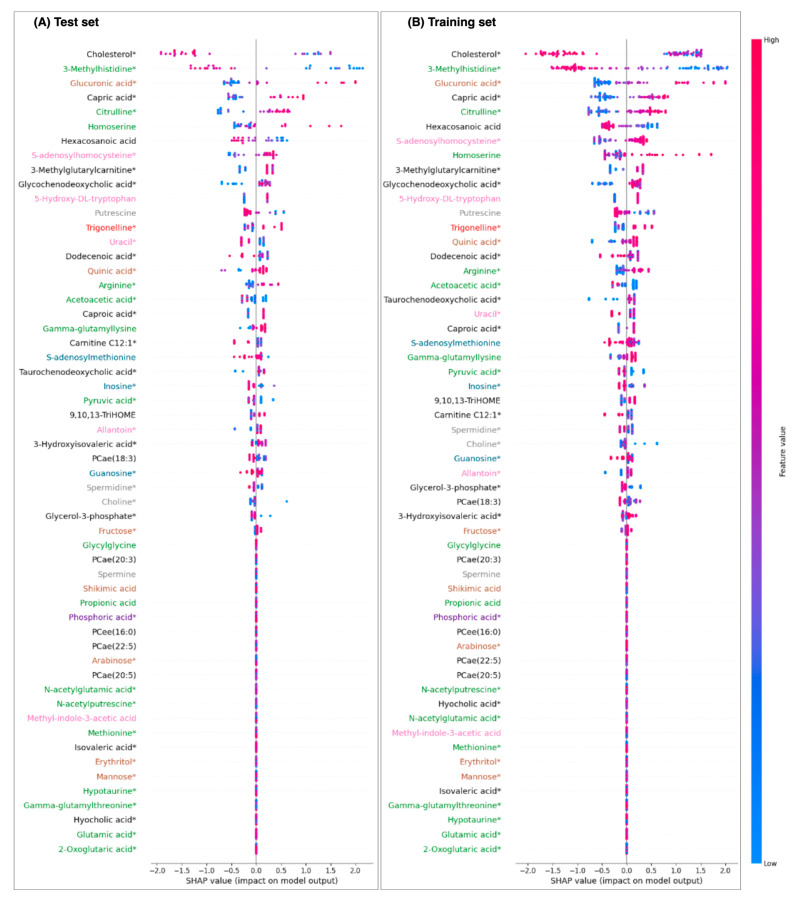
Metabolites’ superclass-informed Shapley analysis (SHAP) of the eXtreme Gradient Boosting model in the FLEMENGHO cohort with the 56 selected features. Positive SHAP values are positively associated with the ASCVD classification. Metabolites marked with * represent those that are also available in the external validation dataset. The colors of the metabolites correspond to their superclasses, as shown in Figure 1.

The SHAP beeswarm plots’ patterns (Figure 2) were very similar for both the training and test sets derived from the FLEMNGHO cohort. In this analysis, lower levels of metabolites like cholesterol, 3-methylhistidine, hexacosanoic acid, and putrescine were associated with a higher likelihood of classification as ASCVD. Conversely, for metabolites such as glucuronic acid, capric acid, citrulline, homoserine, and S-adenosylhomocysteine, elevated levels were positively correlated with the cases. Notably, each of the top three ranked metabolites—cholesterol, 3-methylhistidine, and glucuronic acid—belonged to a distinct superclass, indicating a diverse metabolic impact. Additionally, many of these top-ranking metabolites presented lower cumulative correlations, as reflected by their node areas, underscoring their unique roles in the classification model (Figure 1B).

We observed that several metabolites had a SHAP value of zero (Figure 2), indicating they did not significantly contribute to the XGBoost model’s predictive power when evaluated together with other features. Interestingly, many of the lower-ranked metabolites were highly correlated with one another and clustered within the central region of the correlation network (Figure 1B). This suggests that these metabolites likely provided redundant information, adding little novel value to the model. Notably, phosphoric acid demonstrated no impact (zero SHAP value) and was most strongly correlated with cholesterol, which emerged as the primary feature, further underscoring the limited influence of highly correlated metabolites.

### 2.4. XGBoost Superclass-Specific Modeling of the Training Cohort

**Figure 3:**
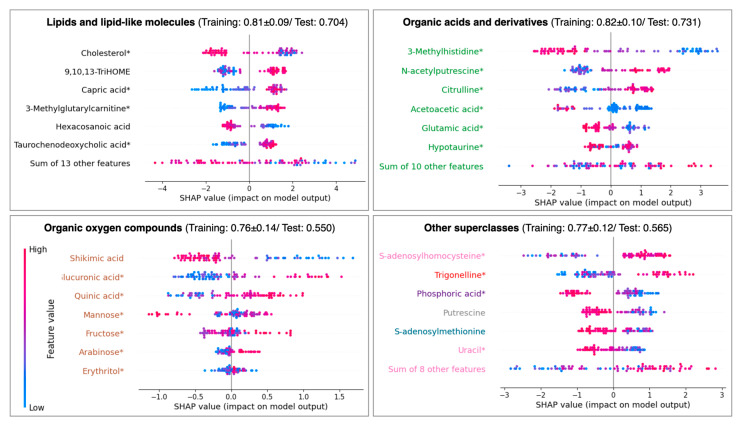
**Shapley analysis (SHAP) of eXtreme Gradient Boosting per the metabolite’s superclass in the training FLEMENGHO set.** Positive SHAP values are positively associated with the ASCVD classification. Other superclasses in the panel include organoheterocyclic compounds (pink); organic nitrogen compounds (grey); nucleosides, nucleotides, and analogues (blue); homogeneous non-metal compounds (purple); and alkaloids and derivatives (red). The values in the subtitles correspond to the weighted ROC AUC during cross-validation of the training set and after hyperparameter optimization of the test set. Metabolites marked with * represent those that are also available in the external validation dataset.

The superclass-specific models for lipids and organic acids, while yielding slightly lower performance compared to the general model, demonstrated the importance for ASCVD discrimination (Figure 3). In the lipids-specific model, cholesterol and capric acid emerged as the top features, consistent with their impacts in the general model. Despite having a relatively minor role in the general model, 9,10,13-TriHOME displayed substantial importance in the lipids-specific model.

A similar trend was observed in the organic acids-specific model. Both 3-methylhistidine and citrulline ranked as top features in both the general and organic acids-specific models. N-acetylputrescine, however, was identified among the top features only in the organic acids-specific model. Other superclasses did not show a good performance in the test set, despite achieving a cross-validation ROC AUC above 0.75 for the training set, suggesting limitations in their predictive capability when isolated from the broader model context.

### 2.5. External Validation Results

In the external validation cohort (MetaCardis), we analyzed 162 control participants alongside 173 patients with IHD. Compared to controls, the IHD group was older and had a higher average BMI, although the sex distribution remained similar. Statistically significant differences in medication use were observed between groups, with a greater proportion of IHD patients on anti-hypertensive and lipid-lowering (statins) medications (Table 2).

Since the MetaCardis and FLEMENGHO cohorts came from independent studies, there was a discrepancy in the metabolites available for analysis in each dataset. Specifically, MetaCardis did not have access to all 56 metabolites selected by PLS-DA in FLEMENGHO. As a result, only a subset of 39 common metabolites was included in the analysis of the MetaCardis cohort.

The external validation results supported the transferability of the identified metabolites from the FLEMENGHO cohort to the MetaCardis cohort. After tunning the XGBoost model, the ROC AUC and F1-score of the test set were 0.929. Given that cholesterol was among these key metabolites and that lipid-lowering medication use was significantly different between groups, we tested a modified model excluding cholesterol. This model’s performance was slightly lower but remained robust (test set ROC AUC/F1-score: 0.843), indicating that cholesterol’s influence, while significant, was not solely responsible for the model’s predictive strength.

## 3. Discussion

This study aimed to leverage explainable ML and metabolomic domain categorization to identify and examine metabolites that distinguish ASCVD cases from controls within the FLEMENGHO cohort. Our findings revealed that lipids and organic acids were the most prominent superclasses, consistently demonstrating strong performance both within their respective groups and in combination with other metabolites. These superclasses, through their diversity and distinct roles, contributed significantly to the model’s capacity to differentiate between cases and controls. Our results were confirmed in an external validation cohort, reinforcing the robustness and transferability of the metabolite-based model developed in this study. The validation demonstrated that the key metabolites identified in the FLEMENGHO cohort retained their discriminatory power in an independent dataset, underscoring their potential applicability across diverse populations.

In our study, within the lipids superclass, fatty acids and glycerophospholipids emerged as the most abundant metabolites distinguishing cases from controls. Fatty acids are known to impact CV disease through various mechanisms, including influencing cardiac metabolism, contributing to lipotoxicity, altering the electromechanical properties of cardiomyocytes, and modulating inflammation [15]. These multifaceted roles underscore the significant influence of fatty acids on ASCVD risk and progression, reinforcing their importance as key metabolites in our analysis.

In our analysis, SHAP values highlighted two key lipids: cholesterol and capric acid. For cholesterol, lower levels were unexpectedly associated with ASCVD, which contrasts with its traditional role as a risk factor for metabolic conditions and CV disease [16,17,18]. This paradox may be explained by the complex interactions within our dataset, particularly the influence of lipid-lowering treatments that could artificially lower cholesterol levels in patients with existing CV disease. On the other hand, while a high cholesterol level is generally linked to an increased risk of ASCVD, some studies suggest exceptions to this association. For instance, Ravnskov [19] noted cases where elevated total cholesterol levels were protective against ASCVD. Similarly, research by Bae et al. [20] and Turusheva et al. [21] identified a U-shaped relationship between total cholesterol levels and mortality, suggesting that both low and high cholesterol levels could be associated with adverse outcomes. In addition, our analysis lacked detailed lipid profile data to distinguish between low- and high-density lipoproteins (LDL and HDL). This distinction is critical, as low levels of HDL are strongly associated with an elevated risk of coronary heart disease and other CV conditions [22].

In our analysis, higher SHAP values for capric acid, also known as decanoic acid, were positively associated with the presence of ASCVD. This finding contrasts with earlier studies, where capric acid and other short- to medium-chain saturated fatty acids (SFAs) showed no significant association with the IHD risk [23]. In another study by Praagman et al. [24], capric acid was grouped with other short-chain SFAs, such as butyric, caproic, and caprylic acids, due to their low dietary intake, and this composite was associated with a slightly reduced IHD risk. In a recent study involving 10,197 men, Oravilahti et al. [25] demonstrated that lower levels of capric acid were inversely associated with mortality, with CV disease accounting for 25% of the observed deaths. Given these mixed findings, more research is needed to clarify the direct impact of specific circulating levels of capric acid on the CV disease risk in humans.

In the organic acids superclass, 3-methylhistidine emerged as the most significant metabolite, with lower values being positively associated with ASCVD. Elevated serum levels of 3-methylhistidine are generally associated with better lean muscle mass and overall nutritional status, and low levels of this metabolite have been identified as a strong, independent predictor of CV events, particularly in patients undergoing maintenance hemodialysis [26]. However, the findings are also mixed. Kouzu et al. [27] reported that higher 3-methylhistidine levels were linked to lower event-free survival rates in patients with symptomatic heart failure, suggesting a potentially complex role of this metabolite in the CV risk across different patient populations. This discrepancy highlights the need for further research to clarify how 3-methylhistidine levels impact the CV disease risk, potentially by differentiating patient subgroups or accounting for underlying health conditions.

While the lipids- and organic acids-specific models performed well in our analysis, the diversity of the most impactful features identified in SHAP analyses suggests that the general model benefited from a broader array of metabolites across different superclasses. This diversity likely enabled the general model to capture complex molecular interactions that superclass-specific models alone may miss. By integrating metabolites from various superclasses, the general model achieved a more nuanced differentiation between cases and controls, indicating that a holistic approach is advantageous for accurately modeling the underlying biochemical distinctions associated with ASCVD. For example, gluconic acid, categorized under the organic oxygen compound superclass (i.e., not a lipid nor an organic acid), was shown to be associated with conditions like hypertension and ischemic stroke and has also been linked to social determinants of health [28]. In addition, Lu et al. [29] also identified gluconic acid as a core metabolite in a multiclass diagnostic model for IHD.

Moreover, examining the model’s results and SHAP values alongside the correlation network suggests that isolating a single metabolite as a reliable biomarker for ASCVD discrimination is challenging. The intricate web of metabolic interactions implies that a multi-metabolite approach may be more effective, leveraging complementary information from various metabolites to enhance classification performance. This complexity also raises important questions about how targeted interventions to modify specific metabolite levels might influence these interdependent relationships and, consequently, the performance of classification models. Adjusting one metabolite could have cascading effects on others, potentially altering the overall metabolic profile and impacting the disease prediction, underscoring the need for an integrated perspective in biomarker development and therapeutic strategies.

In cardiology, efforts to enhance the explainability of ML models have included methods such as class activation maps for image analysis [30] and SHAP values for survival analysis [31]. Within the realm of metabolomics and ASCVD-related data, the application of SHAP has been relatively limited. Existing studies have utilized SHAP to uncover key features influencing ML models, including the cholesterol efflux capacity [32], dilated cardiomyopathy [33], hypertension [34], and ASCVD risk in woman-specific datasets [35]. Our approach not only contributes to extending the use of explainable ML in metabolomics for ASCVD classification but also introduces an innovative integration of metabolite categorization with SHAP visualizations. This additional layer of information enriches the interpretability of the analysis. Since SHAP is model agnostic, this approach can readily be applied to alternative ML models beyond XGBoost in future research.

Metabolomic data are high-dimensional, making it crucial to avoid overfitting in ML models. Therefore, testing models on multiple datasets from different populations can confirm the robustness and generalizability of the ML insights. In our study, the strong results achieved in the external MetaCardis cohort validated our methodology for identifying potential metabolite biomarkers for ASCVD detection. Although IHD is a subset of ASCVD, 52.4% of cases in the FLEMENGHO cohort were identified as IHD-specific. As a result, features associated with the broader ASCVD were applied to a more specific condition, such as coronary atherosclerosis. Interestingly, the MetaCardis model outperformed the model based on the FLEMENGHO cohort. This difference in performance may be attributed to several factors: the larger sample size in MetaCardis, the selection of only patients with IHD, and the pronounced differences between cases and controls in the validation cohort. These included higher medication usage and a greater prevalence of CV-related risk factors, which likely enhanced the model’s capacity to differentiate cases from controls. In this sense, further studies with more homogenous cohorts (for instance, an IHD-specific cohort or those with matched characteristics) are needed to validate this metabolomic signature.

## 4. Limitations

The present study must be interpreted within the context of its limitations and strengths. Regarding the training FLEMENGHO cohort, apart from being a case–control study, ASCVD cases included a mix of atherosclerotic events such as IHD, revascularization, peripheral arterial diseases, and cerebrovascular diseases, which might have different metabolite affinities. The FLEMENGHO cohort had a relatively small sample size, which limited the study’s statistical power and conducting subgroup analyses. The relatively small training cohort size may have led to the identification of certain features that are specific to the sample rather than generalizable to broader populations.

Due to discrepancies in available metabolites between the FLEMENGHO training cohort and the external validation MetaCardis cohort, a subset analysis was conducted within the FLEMENGHO cohort using only the metabolites that matched across both datasets. After hyperparameter optimization, the model achieved a mean ROC AUC of 0.84 ± 0.09 in the FLEMENGHO cohort. In the test set, the model’s ROC AUC decreased to 0.68, with an associated F1-score of 0.69. This adjustment allowed for a fairer performance comparison between cohorts but led to a slight reduction in model performance.

The reduced model performance for the training cohort using only the simultaneously available metabolites highlights the potential impact of excluding some metabolites initially included in the training FLEMENGHO cohort, suggesting that certain metabolites may have played a significant role in enhancing the original classification strength. Nonetheless, the model still demonstrated robust predictive ability with the subset of shared metabolites, showing that the core model was largely generalizable, despite these adjustments. Future studies should focus on validating models in diverse cohorts with standardized measurement protocols to better evaluate the generalizability.

Although we did not perform specific adjustments for medication use, we acknowledge this as a limitation. Statins are known to lower cholesterol-related metabolites, while antihypertensive drugs may affect pathways related to vascular and renal function, potentially impacting metabolomic profiles. The observed associations might partly reflect the metabolic effects of these drugs rather than solely underlying disease processes. For example, the strong associations with cholesterol-related metabolites could be amplified by the widespread use of statins among cases.

Finally, while SHAP provides valuable insights into feature importance by explaining how each metabolite contributes to the model’s predictions, it is inherently correlational, not causal. SHAP shows how variations in specific metabolites affect the output of the classification model but does not reveal whether those metabolites are directly involved in the biological mechanisms of ASCVD. Moreover, further studies that consider different ASCVD events separately could also compare and evaluate the generalizability of our findings.

## 5. Methods

### 5.1. Study Population

The training cohort was derived from a general population cohort from northern Belgium (FLEMENGHO: Flemish study on Environment, Genes and Health Outcomes) [36] The FLEMENGHO study received ethical approval from the Ethics Committee of the University of Leuven (S64406, S67011). In the current study, a subset from the FLEMENGHO cohort that had metabolomic data available was used in a retrospective case–control scenario. We identified 63 patients with a history of ASCVD or evidence of advanced atherosclerotic lesions and 52 non-smoking controls matched by age, sex, and body mass index (BMI) from the same population.

External validation cohort—Open access metabolomic data from the MetaCardis project (https://www.metacardis.net/, accessed on 15 May 2024) was used to derive the external validation cohort [37]. It included 162 healthy controls and 173 patients with IHD, aged 18 to 75, recruited from Denmark, France, and Germany between 2013 and 2015. IHD cases included patients with a first occurrence of acute coronary syndrome, patients with chronic IHD and normal heart function, and patients with IHD and heart failure, as confirmed by echocardiography showing a left ventricular ejection fraction below 45%. The control group was matched with cases by sex and age (within 5 years match).

### 5.2. Metabolomic Profiling

For the training cohort, metabolomic data were acquired using UHPLC 1290/Triple Quadrupole Agilent 6470, GC-Triple Quadrupole 7000C Agilent HP5MS, and UHPLC U3000 Dionex/q-Exactive Orbitrap Thermo Reverse Phase systems following quality control steps. The mass spectrometer was set to automatic tune, and chromatography followed a daily check for analytical suitability and carryover control.

To evaluate sample stability and analytical drifts, non-specific interactions were removed by multi-injections of the quality control pooled sample pre-acquisition, and a repeated quality control pooled sample was injected to monitor and correct the batch. After that, 250 metabolites were available for analysis. For each of them, the area was log2 transformed and centered on the mean of the samples.

The process to acquire metabolomic data from the external validation cohort was described elsewhere [37]. In their study, metabolomic profiling was performed using proton nuclear magnetic resonance, gas chromatography coupled to mass spectrometry, targeted UPLC–MS/MS, and untargeted UPLC–MS.

### 5.3. Metabolite Data Preprocessing and Selection

**Figure 4:**
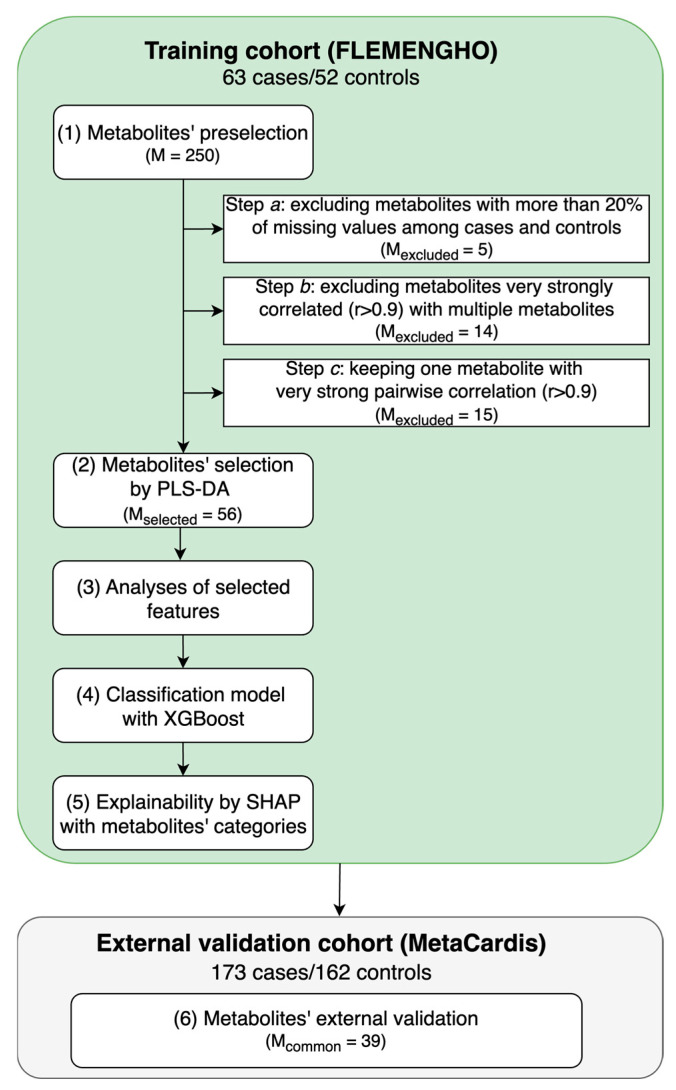
**Analysis pipeline.** In the training cohort (FLEMENGHO), we first identified relevant metabolites to distinguish between atherosclerotic cardiovascular disease (ASCVD) cases and controls. The metabolites were selected from Partial Least Squares Discriminant Analysis (PLS-DA) and then used in eXtreme Gradient Boosting (XGBoost). Next, explainable machine learning of Shapley values (SHAP) with metabolites’ categorization was explored. After that, in an external cohort, we evaluated the same metabolites to distinguish between ischemic heart disease (IHD) cases and controls. In the figure, M stands for the number of metabolites.

For the analysis, we built the computational pipeline illustrated in Figure 4. R 4.4.1 was used for PLS-DA (*mdatools)* and Python 3.11.5 for missing value imputation (*miceforest*), XGBoost model training (*scikit-learn* and *xgboost*) and explainability (*networkx* and *shap*). The scripts related to these analyses are publicly available at https://github.com/HCVE/ascvd_metabolomics, accessed on 29 October 2024.

The first step of the pipeline was metabolites’ preselection. In Step (1*a*), metabolites with more than 20% of missing values both in controls and in ASCVD cases were removed following the “modified 80% rule” [38]. To avoid redundant predictors and multicollinearity problems, in Step (1*b*), metabolites that were very strongly correlated with multiple biomarkers (absolute Pearson’s or Spearman’s correlation > 0.9 with 2 or more variables) were also removed [39]. For the remaining pairs of metabolites whose absolute Pearson’s or Spearman’s correlation was >0.9, one metabolite was kept for analyses (Step 1*c*). A high cutoff was used to not exclude variables that might be similar but that carry different information and might also be involved in distinct interactions.

Missing values were imputed with Multiple Imputation by Chained Equations (MICE) per class. MICE uses chained equations to iteratively fill in missing data by modeling each feature with missing values as a function of the others [40]. Five multiple imputations were averaged to obtain the final one.

PLS-DA, a classical method for dimensionality reduction, was used to select key metabolites associated with ASCVD. PLS-DA is widely applied in biological and chemical data analysis, and it combines dimensionality reduction and discrimination by projecting data onto components maximizing variance and class separation [41]. The PLS-DA model was built using 5-fold stratified cross-validation to ensure the stability of the results. Data were centered and scaled, and the optimal number of latent factors was determined automatically using Wold’s criterion [42], testing up to 10 latent factors. This criterion compares the Predictive Residual Sum of Squares (PRESS) values between successive components, selecting the number of latent factors when the ratio of PRESS for the current component to the next one indicates minimal improvement in the predictive ability. A variable importance in projection (VIP) threshold of 1.2 was used to select the most discriminant metabolites [43].

### 5.4. Domain Knowledge Categorization

The distribution of the selected metabolites was analyzed in terms of their superclass categorization, which groups the metabolites according to general composition or shape [44]). For simplicity, some superclasses will be mentioned in a short version throughout the text (“lipids and lipid-like molecules” will be referred to as “lipids”, “organic acids and derivatives” as “organic acids”, and “alkaloids and derivatives” as “alkaloids”). Information was retrieved based on the Human Metabolome Database (HMDB) [45].

We also analyzed the metabolites’ interactions via a correlation network. The nodes corresponded to the selected metabolites and the edges’ thicknesses depended on the strength of their Spearman’s pairwise correlations. Nodes were placed using the Fruchterman–Reingold force-directed algorithm.

### 5.5. Machine Learning Modeling and Explainability

After dimensionality reduction with PLS-DA, XGBoost [46] was used to assess the validity of the selected features to distinguish ASCVD cases and controls from the FLEMENGHO cohort with a non-linear approach since it is a tree-based ensemble method. The dataset was split into training (75%) and test (25%) sets. The training set was used to compute the centering and scaling parameters, which were then applied to both the training and test sets. Similarly, the imputation was applied to the test set using the parameters derived from the training set.

To optimize the XGBoost model, a grid search was conducted using 5-fold stratified cross-validation of the training set. The model was optimized for the weighted area under the receiver operating characteristic curve (ROC AUC). The hyperparameters included in the grid search are presented in Supplemental Table S1.

Once the best set of hyperparameters was identified, the XGBoost model was retrained on the entire training set using these optimized settings. The final model was then applied to the test set to evaluate its predictive performance on unseen data. Model performance was assessed through weighted ROC AUC and F1-score.

Following the training and evaluation of the XGBoost model, the SHAP analysis was applied, aiming at explaining the model’s predictions and understanding the contributions of individual metabolites to the classification outcomes. SHAP beeswarm plots were generated to visualize the distribution of SHAP values across all samples in the test and training sets. Each feature’s mean absolute SHAP values were computed and ranked on the *y*-axis based on their contributions to the model’s predictions. Features with higher mean SHAP values were considered more influential in driving the association with ASCVD. The values on the *x*-axis represent the contribution of each feature to the prediction for each sample, with positive values indicating a positive impact on predicting ASCVD cases.

To provide deeper insights into the metabolic features associated with ASCVD, we added a modification to SHAP by aggregating metabolites’ superclass information to the same plot in the form of color code. Besides deriving a single model considering all selected features, we also built separate models considering the metabolites’ superclasses.

### 5.6. External Validation

We investigated the validity of the metabolites identified in the training FLEMENGHO cohort using the MetaCardis cohort. The same modeling strategy for training and evaluating XGBoost was used, allowing us to obtain optimal hyperparameters for the external validation dataset, and the performance was evaluated in terms of the weighted ROC AUC and F1-score of the test set.

### 5.7. Statistical Analysis

For statistical analyses, we used Python 3.11 (*scipy* and *statsmodels* libraries). Comparisons between groups were performed using a two-sided t-test for the means of two independent samples (in the case of continuous variables) or the z-test for proportions (in the case of binary variables), with a significance level of 0.01.

## 6. Conclusions

The combination of different metabolites has the potential to build classifiers for ASCVD. Integrating metabolite categorization within the SHAP analysis further enhanced the interpretability of the model, offering insights into metabolite-specific contributions to the ASCVD risk. Thus, integrating metabolomics domain knowledge with explainable ML techniques holds significant promise in advancing the understanding and management of ASCVD. The integration of metabolite categorization facilitated a more structured and interpretable analysis, making it easier to contextualize the roles and relative significance of different metabolite groups in ASCVD discrimination. Future work should continue to address the challenges in data quality, interpretability, and clinical validation to fully realize the benefits of this integrative approach in precision medicine for CV disease. In addition, these findings require further confirmation and validation in larger prospective population studies where the effects of specific demographics, anthropometrics, and medication characteristics on the output might be minimized as much as possible.

## Figures and Tables

**Table 1:** Clinical characteristics of the training cohort (FLEMENGHO).

Characteristics		Controls (N = 52)	Cases (N = 63)	*p*-Value
*Anthropometrics*				
	Age, years	62 ± 8	66 ± 8	0.01
	Females, n (%)	21 (40.4)	19 (30.2)	0.25
	BMI, kg/m^2^	26.3 ± 3.8	27.2 ± 2.8	0.16
	Estimated lean body mass, kg	48.4 ± 9.9	48.4 ± 8.7	1.00
	Systolic blood pressure, mmHg	139.7 ± 18.6	139.4 ± 21.2	0.94
	Diastolic blood pressure, mmHg	84.7 ± 9.5	80.7 ± 10.5	0.03
*Medications*				
	Anti-hypertensive drugs, n (%)	15 (28.8)	37 (58.7)	**0.001**
	Beta-blockers, n (%)	4 (7.7)	17 (27.0)	0.01
	Calcium channel blockers, n (%)	7 (13.5)	13 (20.6)	0.31
	ACE/ANGR1 blockers, n (%)	9 (17.3)	19 (30.2)	0.11
	Diuretics, n (%)	3 (5.8)	16 (25.4)	**0.005**
	Statins, n (%)	12 (23.1)	26 (41.3)	0.04

Data are presented as means ± standard deviations or numbers of subjects (%). Abbreviations: body mass index (BMI); angiotensin-converting enzyme (ACE); angiotensin receptor type 1 (ANGR1). *p*-values lower than 0.01 are bolded.

**Table 2:** Clinical characteristics of the external validation cohort (MetaCardis).

Characteristics		Controls (N = 162)	Cases (N = 173)	*p*-Value
*Anthropometrics*				
	Age, years	58 ± 10	61 ± 8	**0.003**
	Females, n (%)	102 (63.0)	119 (68.8)	0.26
	BMI, kg/m^2^	22.6 ± 1.8	28.3 ± 5.4	**<0.001**
	Systolic blood pressure, mmHg	127.8 ± 15.9	127.4 ± 15.8	0.82
	Diastolic blood pressure, mmHg	70.3 ± 9.4	69.9 ± 9.7	0.70
*Medications*				
	Anti-hypertensive drugs, n (%)	13 (8.0)	158 (91.3)	**<0.001**
	Beta-blockers, n (%)	5 (3.1)	140 (80.9)	**<0.001**
	Calcium channel blockers, n (%)	1 (0.6)	31 (17.9)	**<0.001**
	ACE/AT2 inhibitors, n (%)	6 (3.7)	100 (57.8)	**<0.001**
	Diuretics, n (%)	1 (0.6)	67 (38.7)	**<0.001**
	Statins, n (%)	8 (4.9)	161 (93.1)	**<0.001**

Data are presented as means ± standard deviations or numbers of subjects (%). Abbreviations: body mass index (BMI); angiotensin-converting enzyme (ACE); angiotensin type 2 (AT2). *p*-values lower than 0.01 are bolded.

## Data Availability

The original contributions presented in the study are included in the article and Supplementary Materials; further inquiries can be directed to the corresponding author.

## Supplementary Materials

The following supporting information can be downloaded at: https://www.mdpi.com/article/10.3390/ijms252312905/s1.
