# Supplementary material for: Integrating Metabolomics Domain Knowledge with Explainable Machine Learning in Atherosclerotic Cardiovascular Disease Classification
Source: Int J Mol Sci. 2024 Nov 30;25(23):12905. doi: 10.3390/ijms252312905 (PMC11641503; doi:10.3390/ijms252312905)
Supplement: Supplementary file 1 [file ijms-25-12905-s001.zip › ijms-3316997-supplementary.pdf]

Supplements

Supplemental Table S1: Hyperparameters search space for eXtreme Gradient Boosting.

Hyperparameter	Description	Experimental search space
n_estimators	Number of trees	[50, 100, 150]
max_depth	Maximum depth of each tree	[3, 6, 9]
eta	Learning rate	[0.05, 0.1, 0.3, 0.6]
subsample	Subsample ratio of the training instance	[0.9, 1]

Supplemental Table S2: Metabolites missing data frequency.

	Controls	Cases
Carnitine C12:0	28	33
13-HOTrE	25	24
Prostaglandin-F1a	24	31
9-cis 13-cis-methyl-retinoate	19	37
Cytidine	19	17
9-oxo-ODE	13	8
Hydroxyphenyllactic acid	11	9
Thromboxane-B2	9	4
PCae(22:3)	8	12
Choline phosphate	7	4
Methylmalonic acid	5	2
Dodecanedioic acid	4	4
S-adenosylhomocysteine	5	8
3-phenylpropionic acid	3	6
Adenine	3	5
9(S)-HODE	3	0
3-hydroxy-DL-kynurenine	2	4
Eicosenoic acid	2	1
Hydroxydodecanoic acid	1	4
Indoxyl sulfate	1	2
Caproic acid@9.79-qpneg	1	1
Hexacosanoic acid	1	1
N-acetylglutamine	1	1
Ribose-5-phosphate	1	1
Guanosine	0	2
Taurine	0	1
Stearic acid	0	2
Spermine	0	1
Spermidine	0	1

	**Controls**	**Cases**
S-adenosylmethionine	0	4
Putrescine	0	1
Benzoic acid	0	1
Heptadecatrienoic acid	0	1
PCae(20:3)	0	2
PCae(12:0)	0	1
Orotic acid	0	1
Ornithine	0	1
N8-acetylspermidine	0	1
N1-acetylspermidine	0	1
N-acetylputrescine	0	1
N-acetylglutamic acid	0	1
Carnitine C5:0	0	1
PCae(20:5)	0	3

Supplemental Table S3: Metabolites excluded before analyses.

**Step**	**Metabolites excluded**
80% rule exclusion	Prostaglandin-F1a<br>9-cis 13-cis-methyl-retinoate<br>13-HOTrE<br>Cytidine<br>Carnitine C12:0
Multiple very strongly correlated	5-aminovaleric acid<br>Carnitine C14:2<br>Heptadecenoic acid<br>2-oxovaleric acid<br>2-methylbutyric acid<br>12(S)-HETE<br>Linoleic acid<br>9(S)-HODE<br>Ketoisocaproic acid<br>Palmitic acid<br>4-hydroxy-phenyllactic acid<br>Ketoisovaleric acid<br>Isobutyric acid<br>9-oxo-ODE
Pairwise very strongly correlated	3-hydroxybutyric acid<br>Indoxyl sulfate<br>4-hydroxymandelic acid<br>Alpha-tocopherol<br>Caprylic acid<br>Carnitine C10:0<br>Carnitine C16:0<br>Eicosenoic acid<br>Gamma-glutamylleucine<br>Hydroxyphenyllactic acid<br>N8-acetylspermidine<br>PCae(15:0)<br>PCae(18:0)<br>Ribose<br>Tyrosomuricholic acid

Supplemental Table S4: Metabolites preselected PLS-DA with VIP>1.2. Metabolites marked with * represent those available also in the external validation dataset.

**Metabolites**
2-oxoglutaric acid*
3-hydroxyisovaleric acid*
3-methylglutarylcarnitine*
3-methylhistidine*
5-hydroxy-DL-tryptophan
9,10,13-TriHOME
Acetoacetic acid*
Allantoin*
Arabinose*
Arginine*
Capric acid*
Caproic acid*
Carnitine C12:1*
Cholesterol*
Choline*
Citrulline*
Dodecenoic acid*
Erythritol*
Fructose*
Mannose*
Gamma-glutamyllysine
Gamma-glutamylthreonine*
Glucuronic acid*
Glutamic acid*
Glycerol-3-phosphate*
Glycochenodeoxycholic acid*
Glycylglycine
Guanosine*
Hexacosanoic acid
Homoserine
Hyocholic acid*
Hypotaurine*
Inosine*
Isovaleric acid*
Methionine*
Methyl-indole-3-acetic acid
N-acetylglutamic acid*
N-acetylputrescine*
PCae(18:3)
PCae(20:3)

PCae(20:5)<br>PCae(22:5)<br>PCee(16:0)<br>Phosphoric acid*<br>Propionic acid<br>Putrescine<br>Pyruvic acid*<br>Quinic acid*<br>S-adenosylhomocysteine*<br>S-adenosylmethionine<br>Shikimic acid<br>Spermidine*<br>Spermine<br>Taurochenodeoxycholic acid*<br>Trigonelline*<br>Uracil*
